# Golgi Fragmentation and Sphingomyelin Transport to *Chlamydia trachomatis* during Penicillin-Induced Persistence Do Not Depend on the Cytosolic Presence of the Chlamydial Protease CPAF

**DOI:** 10.1371/journal.pone.0103220

**Published:** 2014-07-28

**Authors:** Stephanie Dille, Katharina Herbst, Larisa Volceanov, Thilo Nölke, Oliver Kretz, Georg Häcker

**Affiliations:** 1 Institute of Medical Microbiology and Hygiene, University Medical Centre Freiburg, Freiburg, Germany; 2 Institute of Experimental and Clinical Pharmacology and Toxicology, University of Freiburg, Freiburg, Germany; 3 BIOSS Centre for Biological Signalling Studies, University of Freiburg, Freiburg, Germany; Institut Pasteur-URACNRS2582, France

## Abstract

*Chlamydia* grows inside a cytosolic vacuole (the inclusion) that is supplied with nutrients by the host through vesicular and non-vesicular transport. It is unclear in many respects how *Chlamydia* organizes this transport. One model posits that the *Chlamydia*-induced fragmentation of the Golgi-apparatus is required for normal transport processes to the inclusion and for chlamydial development, and the chlamydial protease CPAF has been controversially implicated in Golgi-fragmentation. We here use a model of penicillin-induced persistence of infection with *Chlamydia trachomatis* to test this link. Under penicillin-treatment the inclusion grew in size for the first 24 h but after that growth was severely reduced. Penicillin did not reduce the number of infected cells with fragmented Golgi-apparatus, and normal Golgi-fragmentation was found in a CPAF-deficient mutant. Surprisingly, sphingomyelin transport into the inclusion and into the bacteria, as measured by fluorescence accumulation upon addition of labelled ceramide, was not reduced during penicillin-treatment. Thus, both Golgi-fragmentation and transport of sphingomyelin to *C. trachomatis* still occurred in this model of persistence. The portion of cells in which CPAF was detected in the cytosol, either by immunofluorescence or by immune-electron microscopy, was drastically reduced in cells cultured in the presence of penicillin. These data argue against an essential role of cytosolic CPAF for Golgi-fragmentation or for sphingomyelin transport in chlamydial infection.

## Introduction

A number of chlamydial species infect humans and/or animals. The clinically most relevant human pathogenic species is *Chlamydia trachomatis*, which causes eye infections and infections of the urogenital tract on a massive scale worldwide [1].

*Chlamydiae* are obligate intracellular bacteria that largely infect epithelial cells and that have a unique developmental cycle [2]. They occur in two differentiation states: the elementary body (EB) is small and infectious but has little metabolism [3] while the reticulate body (RB) is larger, metabolically active and divides but is non-infectious. The cycle starts when the EB is taken up by a host cell. The endocytic vacuole is modified by chlamydial proteins and develops into a specialized intracytoplasmic organelle termed inclusion, where upon differentiation of EB to RB the bacteria start to replicate. Eventually RBs differentiate back into EBs, which are released to infect other cells. *In vitro,* this cycle takes about 48 h for *C. trachomatis* [2].

The inclusion provides a protective and optimized environment for the replicating bacteria. However, growth in the inclusion also necessitates the acquisition of nutrients across the inclusion membrane including host-derived membrane lipids (sphingomyelin and cholesterol) as well as phospholipids for the growing inclusion membrane and the bacteria themselves [4]–[7]. In 1995, Ted Hackstadt and colleagues showed that exogenously added ceramide is converted to sphingomyelin within the host cell and taken up into the bacterial cell wall [8]. Since then, many studies have investigated aspects of lipid transport to the chlamydial inclusion (for review see [9]). Part of lipid-acquisition appears to occur by redirection of post-Golgi-trafficking to the inclusion and the fusion of post-Golgi-vesicles with the inclusion membrane [10], [11]. One factor that seems relevant for this route is the infection-associated fragmentation of the Golgi apparatus (GA) [12]. GA-fragmentation coincides with the (contentious) loss of the GA-matrix protein golgin-84, and since the experimental reduction of golgin-84 also causes GA-fragmentation, the infection-induced loss of this protein may be an explanation for the changes to the GA (although it would appear likely that the arrangement around the inclusion requires additional factors). However, a more recent study suggests that the degradation of golgin-84 is at least partly caused by proteolysis during experimental preparation of cell lysates [13], and CPAF-deficient chlamydial strains still show Golgi-fragmentation [14].

We have recently found that golgin-84 can be cleaved during ectopic expression (i. e. expression without infection) of the chlamydial protease CPAF [15]. CPAF enters the host cell cytosol from the inclusion at about 14–16 h after infection with *C. trachomatis*, as evidenced by immunofluorescence [16]. A number of host cell CPAF-substrates are known. These proteins have been found to be cleaved during infection and mostly upon addition of CPAF to cell lysates. Generally, biological consequences have been attributed to these cleavage events although the already mentioned study shows that the extent of cleavage observed can depend on the sample preparation, suggesting that at least a substantial part of the cleavage is an extraction artifact [13].

The potential link between the secretion of CPAF, proteolysis of host cell proteins, fragmentation of the GA and lipid transport to the inclusion is still uncertain (although the mentioned study of CPAF-deficient *C. trachomatis* adds some clarity). The extent of proteolysis in intact cells is unknown, and it is not possible to block GA-fragmentation directly and without side effects. We have in the past used a synthetic tetrapeptide-inhibitor (WEHD-fmk) that clearly appears to block CPAF activity [15], and this inhibitor blocks not only CPAF-dependent effects but also chlamydial growth and GA-fragmentation. However, it is impossible to know whether it also interferes with other bacterial or host factors and may therefore indirectly block GA-fragmentation.

To obtain additional information on the interrelation of the described events we here used a model of chlamydial persistent infection. A ‘persistent’ state of *Chlamydia* can be readily induced by treatment of infected cell cultures with Penicillin G (PenG), IFN-γ or iron chelating agents [17]. *In vitro* persistent infections are characterized by enlarged RBs of atypical morphology, which divide very little (‘aberrant RBs’) [18]. Whether persistence plays a role *in vivo*, such as in chronic infections, is not clear. However, experimental induction of persistence alters a number of features of the infection and therefore offers a possibility to test cell biological aspects of chlamydial infection in a situation that is, although not very well defined, different from acute infection. Three different cell types were in this study infected with *C. trachomatis*, and acute infection was compared to PenG-induced persistence. We compared localization of CPAF, GA-fragmentation and sphingomyelin-transport to the bacteria between these two conditions. Golgi-fragmentation was further tested in one of the described CPAF-deficient strains of *C. trachomatis*.

## Materials and Methods

### Cell culture

All cell lines were cultured at 37°C and 5% CO_2_. HeLa cells and mouse embryonic fibroblasts (MEFs [19]) were maintained in Dulbecco modified Eagle’s minimal essential medium (DMEM) supplemented with 10% fetal calf serum (FCS, tetracycline negative; PAA Laboratories), for the MEF cells, 50 µM β-mercaptoethanol (Gibco) was added. The mouse oviduct epithelial cells (C57epi.1 [20]) were a kind gift of Dr. Raymond Johnson (Division of Infectious Diseases, Indiana University School of Medicine, Indianapolis, Indiana, USA) and were maintained in epithelial growth medium (1∶1 DMEM and Nutrient Mixture F12-Ham (Sigma, #N3520) with 3.1 g/L sodium bicarbonate (Sigma, #S5761), 0.5 mg/ml bovine insulin solution (Sigma, #I4011), 10% FCS Gold (PAA Laboratories), 2 nM Glutamax I (Gibco) and 25 ng/µl Keratinocyte Growth Factor (KGF, Sigma #K1757)).

### Infection with *C. trachomatis*

*Chlamydia trachomatis* LGV2 (L2) was obtained from the American Type Culture Collection (ATCC) and stored in SPG at −80°C. The CPAF nonsense mutant CTL2-M169 [RST17] (CPAF−) and the corresponding CPAF expressing control strain CTL2-M169 [RST5] (CPAF+) were kindly provided by Dr. Raphael H. Valdivia (Department of Molecular Genetics and Microbiology, Center for Microbial Pathogenesis, Duke University Medical Center, Durham, NC, USA). One day prior to infection, cells were seeded in their respective culture medium and incubated at 37°C with 5% CO_2_ overnight. *Chlamydiae* were added directly to the cells at the specified multiplicity of infection (MOI). 100 U/ml PenG (Sigma) was added at the same time as the *Chlamydiae*. PenG concentration was as in a previous report [21].

### Immunofluorescence

For immunofluorescence cells were seeded in 24 well plates onto coverslips and infected as described above. For microscopy cells were fixed in methanol for 10 minutes or in 4% PFA for 20 minutes, permeabilized for 10 minutes in 0.2% Triton X-100 (Sigma) in PBS and blocked in 5% bovine serum albumin (BSA, Sigma) in PBS. Antibodies used were: rabbit anti-*Chlamydia* (1∶2.000, Milan Analytica #20-698); Cy5-donkey anti-rabbit (1∶300, Jackson ImmunoResearch); FITC-donkey anti-rabbit (1∶300, Dianova), mouse anti-CPAF clone 100a (1∶200, kind gift from Dr. Guangming Zhong, Department of Microbiology and Immunology, University of Texas Health Science Center at San Antonio, Texas, USA); Cy5-donkey anti-mouse (1∶300, Dianova). Subsequently the samples were stained with Hoechst (1∶15.000, Sigma) for 10 minutes before being mounted in Permafluor (Thermo Fisher). The samples were analyzed with an AxioPlan 2 microscope (Zeiss) using the AxioVision software 4.8.2 (Zeiss) or with a BZ 9000E microscope (Keyence) using the BZ II Analyzer software 1.42 (Keyence). Images were processed and assembled with Adobe Illustrator CS6 (Adobe).

### Electron microscopy

HeLa cells were seeded in 24 well plates onto coverslips and infected with *C. trachomatis* L2 for 30 h. The cells were fixed in 4% PFA/0, 1% glutaraldehyde (EM grade, Roth) for 20 minutes and stained over night with the mouse anti-CPAF antibody (1∶50) in 50 mM TBS/2% normal goat serum, washed with 50 mM TBS and stained over night with a gold-coupled anti-mouse antibody (1∶100, Nanoprobes). After silver intensification (HQ silver, Nanoprobes) and counterstain with 0.1% osmium tetroxide (Roth), samples were embedded in Durcupan (Fluka), subjected to ultra-thin-sectioning and analyzed with a Zeiss Leo TEM. Quantification of immunogold labeling was performed using ITEM software (Olympus, Germany). Briefly, pictures of infected cells were randomly taken, the regions of interest (inclusions or cytoplasm) were outlined and the number of gold particles was counted within these regions. Non infected, stained cells of the same culture dishes were taken as negative control.

### Ceramide transport

HeLa cells were seeded in glass bottom dishes (MatTek, Ashland, MA) and infected with *C. trachomatis* L2, with or without addition of 100 U/ml PenG, for 24 h. The medium was replaced with fresh medium containing 100 nM BODIPY FL C_5_ ceramide (Invitrogen #B22650). After 30 minutes, cells were subjected to time lapse microscopy at 37°C in a chamber with humidified atmosphere (6.5% CO_2_ and 9% O_2_). An inverted Axiovert 200 M microscope (Zeiss) equipped with a Yokogawa CSU-X1 spinning disc confocal head (Tokyo, Japan), an emission filter wheel, a Coolsnap HQ II digital Camera (Roper Scientific, Tucson, AZ) and driven by Metamorph imaging software version 7.7.11.0 (Universal Imaging) was used to collect images. BODIPY FL C_5_ ceramide was excited with a 488 nm solid state laser. Fluorescence intensities were measured inside the inclusion in a 34 µm^2^ area (acute n = 35; persistent n = 26 inclusions) at the indicated time points. Averages and SEM of two independent experiments were calculated using Microsoft Excel 2010.

### Western Blot

For Western Blot analysis, HeLa cells were seeded in 6 well plates and infected as described above. At the indicated time points, either Urea (8 M) or RIPA-extracts were prepared: for RIPA-extracts, cells were washed with PBS, trypsinized and pelleted (3824 g, 5 min, 4°C). After a washing step with PBS, cells were resuspended in RIPA-Buffer (Sigma) supplemented with Protease Inhibitor (Complete, Roche) and Benzonase (25 U/ml, Novagen). After 30 minutes incubation on ice cell debris was removed (17949 g, 10 min, 4°C) and the supernatant was transferred to a fresh cap. For Urea-extracts, cells were kept on ice, washed with PBS and incubated for 10 min with 8 M urea (J.T. Baker), supplemented with 325 U/ml Benzonase (Novagen). Cells were harvested by scraping, pelleted and the supernatant was transferred to a fresh tube. For both extraction methods, supernatants were then supplemented with 6x Laemmli-Buffer to a final concentration of 1x and heated to 95°C for 10 min. Prior to addition of the SDS-sample buffer, an aliquot was taken for protein assay (Biorad). Ten micrograms protein was loaded onto 10% SDS gels and transferred to nitrocellulose membranes. After blocking the membranes (5% milk in TBS-T) they were incubated with the respective antibody at 4°C overnight. Primary antibodies were directed against chlamydial Hsp60 (mouse, 1∶1.000, Enzo Life Sciences #ALX-804-072), CPAF (rabbit, 1∶1.000, [22]) and β-actin (mouse, 1∶10.000 Sigma #A5441); Secondary antibodies: Goat anti-rabbit (Sigma #A6667), goat anti-mouse (Jackson ImmunoResearch #115-035-166).

### Construction of stable cell lines

In order to construct the stable HeLa cell line YFP-Golgi-HeLa, a fusion protein of enhanced yellow fluorescent protein (EYFP) and the N-terminal 81 amino acids of human beta 1,4-galactosyltransferase (the membrane-anchoring signal peptide that targets the fusion protein to the trans-medial region of the Golgi apparatus [23]) was cloned into the lentiviral vector pFCMV-TO-GW (kind gift from Dr. John Silke, Department of Biochemistry, LaTrobe University, Melbourne, Australia). HeLa cells were lentivirally transduced and selected with 1.5 µg/ml puromycin (Invivogen).

## Results

### Inclusion growth in acute and persistent infection

We used the human epithelial cell line HeLa, a line of spontaneously immortalized mouse oviduct epithelium cells [20] and mouse embryonic fibroblasts (MEFs) for infection with *C. trachomatis* serovar L2. Persistence was induced by addition of 100 U/ml penicillin G (PenG) at the time of infection. The infection rate (inclusions per bacteria added) was only mildly reduced by this treatment but all developing inclusions harbored large aberrant RBs (data not shown).

**Figure 1:**
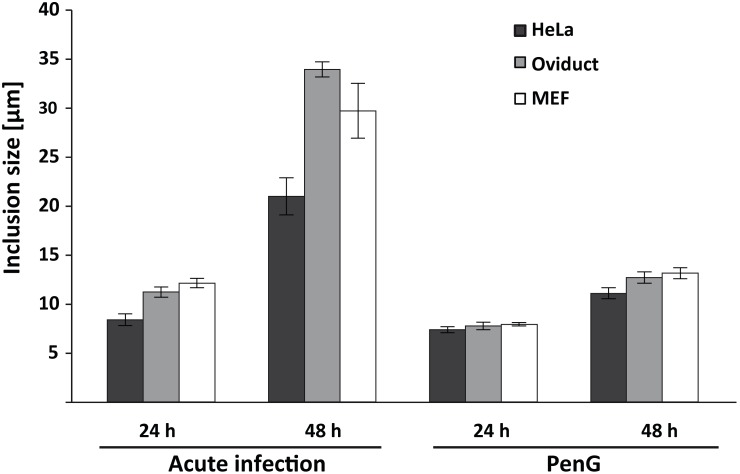
Inclusion size during acute and persistent chlamydial infection and in the presence of peptide inhibitors. HeLa, oviduct epithelial cells and MEFs were infected with *C. trachomatis* L2 at an MOI of 1 with or without addition of 100 U/ml PenG. At 24 or 48 h post infection, cells were fixed, processed for immunofluorescence and inclusion size was measured. Shown are the means of 3 independent experiments ± SEM, 15 view-fields per sample were evaluated using the AxioVision Software (number of inclusions measured = 244, 219, 299, 295 for HeLa; 201, 209, 164, 160 for oviduct and 135, 212, 181, 153 for MEFs). *C. trachomatis* is also inhibited by lower concentrations of PenG [42] but this concentration has been used before [21].

In the presence of PenG the chlamydial inclusion still grew almost to the same size as in acute infection within the first 24 h but then almost stopped growing (Fig. 1; all the raw data used to calculate the results shown in this manuscript are given in Table S1) (this has been observed before [21]). At 24 h p.i. the inclusions in acute (compared to persistent) infection were larger by a factor of 1.14 in HeLa, 1.45 in oviduct and 1.53 in MEF cells. In acute infection the inclusion appears densely packed with *Chlamydia.* In PenG-treated cultures (as observed before [21]) there appeared to be free space unoccupied by bacteria in the inclusions, which contained no more than 4–6 aberrant RBs (not shown).

### GA-fragmentation in acute and persistent infection

GA-fragmentation is now a well-established event of chlamydial infection, and we have found it also during ectopic expression of CPAF [15]. We next tested whether Golgi-fragmentation also occurred in PenG-induced persistence. We first generated a HeLa cell line stably expressing β-1,4-galactosyltransferase fused to YFP. Beta-1,4-galactosyltransfrase localizes to the GA [24], and the fusion to YFP permits easy assessment of GA-structure. This cell line was infected with *C. trachomatis* with or without addition of PenG.

**Figure 2:**
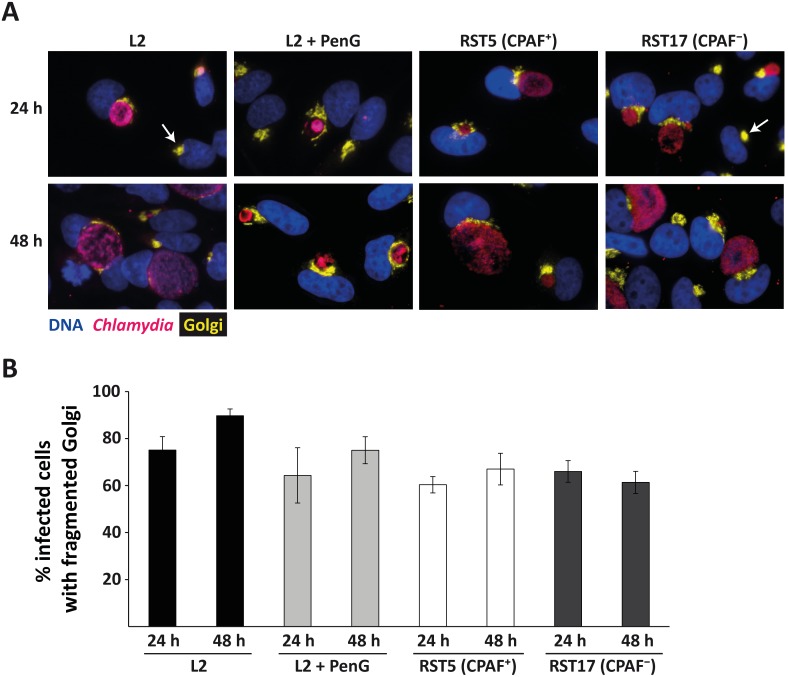
Golgi-fragmentation during acute and persistent infection. (**A**) YFP-Golgi-HeLa cells were infected with *C. trachomatis* L2 with or without addition of 100 U/ml PenG, RST17 (CPAF−) and the corresponding control strain RST5 (CPAF+) [14], incubated for the indicated times and processed for immunofluorescence. Blue: Hoechst DNA-stain, yellow: GA, pink: *Chlamydia*. All images were taken at 40-fold magnification. Arrows point to uninfected cells displaying a normal, not fragmented GA. (**B**) Quantification of the portion of infected cells showing a fragmented GA. All infected cells as well as all infected cells with fragmented GA were counted and the ratio of fragmentation-positive cells was calculated (number of infected cells counted: 499, 629, 478, 517, 397, 475, 380, 522 from left to right). Shown are means of 3 independent experiments ± SEM.

The GA was clearly visible in all cells. During both acute and persistent infection fragmentation of the GA was apparent (Figure 2A). Between about 80% and 95% of infected cells displayed GA-fragmentation. This fraction was slightly higher at 48 h compared to 24 h in both conditions and slightly higher in acute compared to persistent infection although this latter difference was not statistically significant (Fig. 2B). Golgi-fragmentation therefore still occurs in PenG-induced persistence of *C. trachomatis*.

CPAF-deficient mutants have been described recently, which were found to induce Golgi-fragmentation in a way identical or very similar to the wild type strain [14]. We used two of the described mutant strains to confirm these results. The two mutants (RST5 and RST17) differ in that RST17 has a nonsense mutation in CPAF (i.e. can express only a truncated CPAF); on top of this there are only three more nucleotides different [14]. Although the induction of Golgi-fragmentation by these two strains was slightly below our wild type, there was no difference between the two strains (whose main difference is the expression of CPAF), confirming that CPAF is not essential for Golgi-fragmentation during infection with *C. trachomatis* (Fig. 2A, B).

### Sphingomyelin-transport to the inclusion during PenG-induced persistence

Sphingomyelin (SM) is at least in part acquired by *C. trachomatis* in a post-GA-vesicular trafficking pathway [11] although some SM can also be transported through direct transfer from the ER [25], [26]. When fluorescent ceramide is added to infected cells, SM-transport to first the inclusion membrane and then into the bacterial membranes can be visualized. Since this transport occurs in the presence of GA-fragmentation and is likely to be linked to both growth of inclusion membrane and division of the bacteria we measured SM-transport to the inclusion in PenG-induced persistence. Fluorescent ceramide was added to the cultures and transport of the fluorescence signal to the inclusion and into the bacteria was monitored.

**Figure 3:**
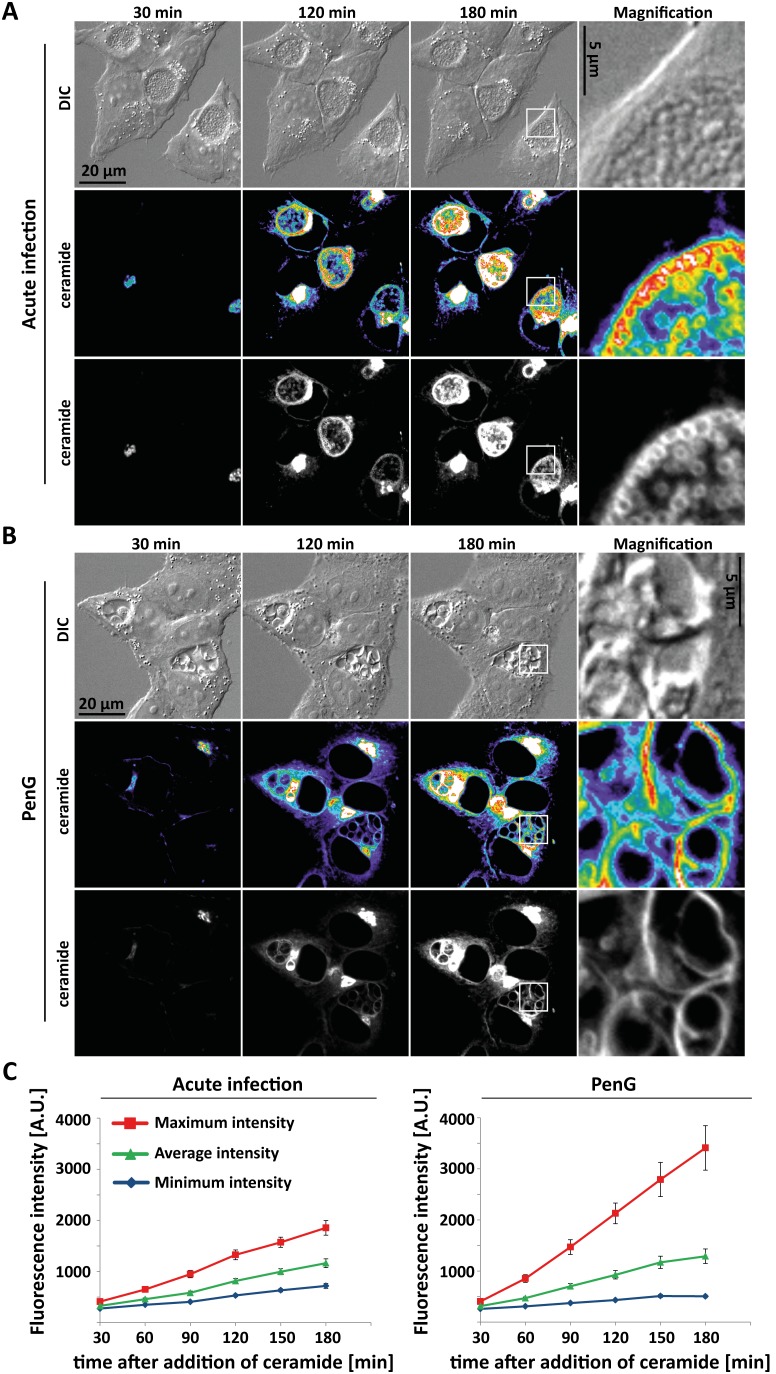
Ceramide transport in acute and persistent infection. Hela cells were infected with *C. trachomatis* L2 at an MOI of 1 for 24 h without (**A**) or with addition of 100 U/ml PenG (**B**). Confocal microscopic movies were recorded and exemplary pictures taken 30, 120 and 180 minutes after addition of BODIPY-FL C_5_ ceramide are shown. The right column gives enlargements of the area indicated by the square. The heatmap ranges from purple (low intensity) to white (high intensity). (**C**) Quantification of fluorescence intensity. Averages of two independent experiments are given ± SEM (35 inclusions in acute and 26 inclusions in persistent infection).

During these experiments, fluorescence can be detected both in the inclusion membrane and, somewhat later, in the bacteria. Since the shape of aberrant bacteria is severely distorted we quantified sphingomyelin-transport by measuring the increase in minimum, average or maximum intensity inside the inclusion upon addition of fluorescently labelled ceramide. As shown in Fig. 3, the increase in average intensity was very similar in acute and persistent infection. The maximum intensity was even stronger in persistent infection, possibly as a consequence of the concentration of the dye in fewer (albeit bigger) individual bacteria. Taken together, the data indicate that the association of GA-fragmentation and sphingomyelin-transport into the inclusion is maintained in persistent infection. Golgi-fragmentation may thus be associated with changes in post-GA transport mechanisms.

### CPAF-expression and secretion in acute and persistent infection

CPAF is produced as a zymogen, processed during its activation and secreted dependent on the bacterial type II-secretion system [27]. How CPAF reaches the cytosol is uncertain. During ‘persistence’ of both *C. pneumoniae* [28] and *C. trachomatis* [29] CPAF-secretion into the cytosol has been reported not to occur.

**Figure 4:**
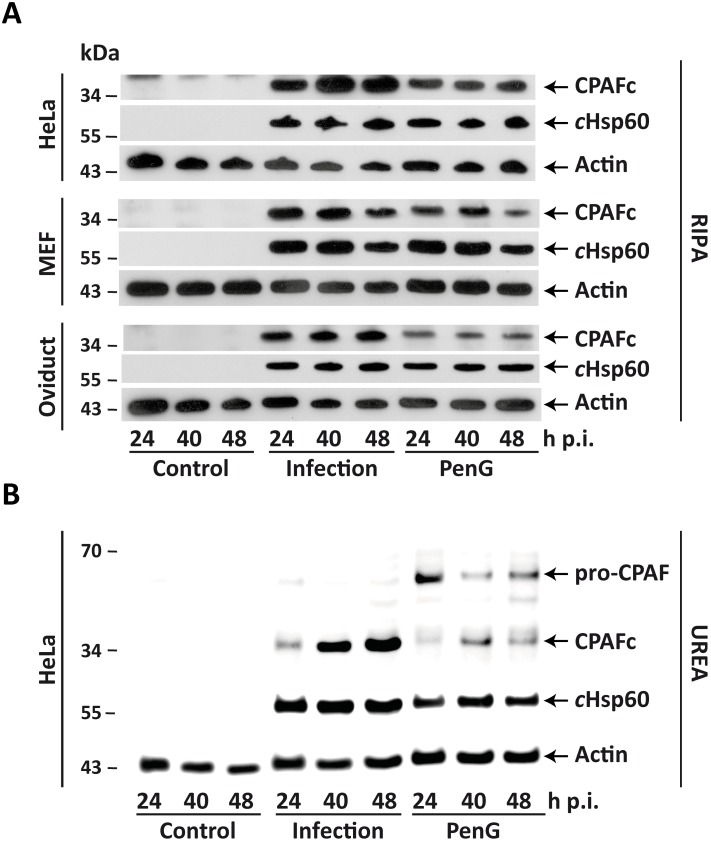
CPAF expression during acute and persistent infection. HeLa, oviduct epithelial cells and MEFs were infected with *C. trachomatis* L2 at an MOI of 1 with or with addition of 100 U/ml PenG. Shown are representative Western Blots of uninfected (Control), acutely infected (Infection) or persistently (PenG) infected cells. Whole cell lysates were prepared with either RIPA (**A**) or UREA (**B**) extraction buffer (see methods) at 24, 40 and 48 h p.i. and 10 µg protein was loaded onto each lane. CPAFc: active CPAF; *c*Hsp60: chlamydial Hsp60 protein; Actin: used as loading control.

Expression of processed (active) CPAF was detectable in all three cell types by Western blotting. PenG clearly reduced the amount of active CPAF but the active protease was still easily detectable in lysates from all cell lines and at all time points tested (Fig. 4). Since substantial CPAF-dependent proteolysis of host cell proteins is known to occur during extraction with detergent [13], we also performed extraction in buffer containing 8 M urea (reported not to permit this extraction-associated cleavage). In detergent extracted lysates only active CPAF was detected (Fig. 4A and not shown), and the same was the case for urea-extracts in acute infection (Fig. 4B) (the latter has been reported before [13]). However, substantial amounts of unprocessed CPAF were detectable in urea-extracts from PenG-treated infected cultures (Fig. 4B). This suggests that PenG-treatment indeed reduces the processing of CPAF and that further artificial processing of CPAF can occur when cultures containing unprocessed CPAF are extracted with detergent.

**Figure 5:**
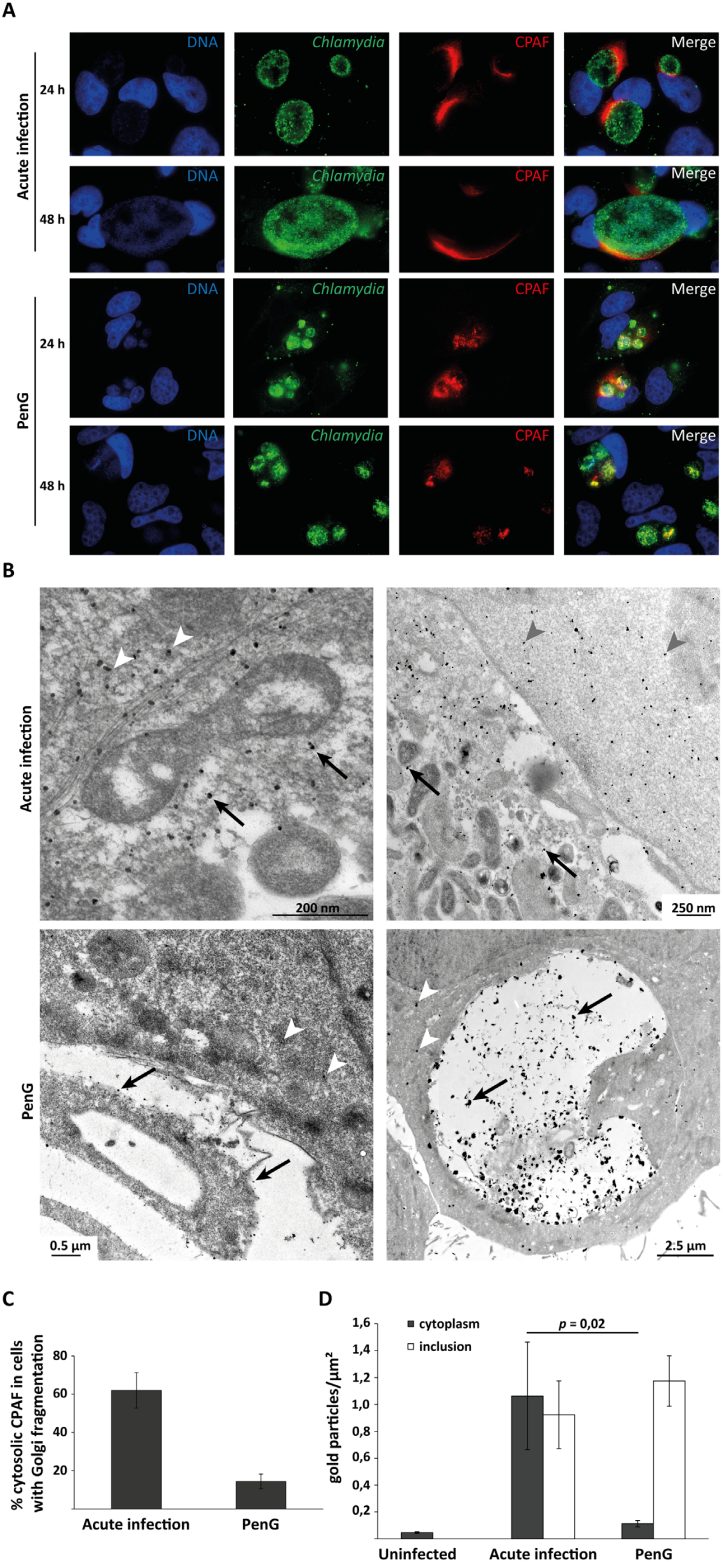
CPAF localisation during acute and persistent infection. (**A**) HeLa cells were infected with *C. trachomatis* L2 at an MOI of 1, with or without addition of 100 U/ml PenG, incubated for 24 or 48 h and processed for immunofluorescence. Blue: Hoechst DNA-stain, red: CPAF, green: *Chlamydia*. All images were taken at 100-fold magnification. For quantification of cell numbers with Golgi-fragmentation and detectable cytosolic CPAF see Fig. 5C. (**B**) Immunogold labelling of CPAF in HeLa cells infected for 30 h with *C. trachomatis* L2 at an MOI of 1 without (upper row) or with addition of 100 U/ml PenG (lower row). Black arrows indicate CPAF inside the inclusions, white or grey arrowheads indicate free CPAF in the cytoplasm. For quantification of the staining see Fig. 5D. (**C**) Relative numbers of infected cells that show cytosolic CPAF as well as Golgi fragmentation in immunofluorescence. YFP-Golgi-HeLa cells were infected for 24 h without or with addition of 100 U/ml PenG, fixed and stained for CPAF and DNA as in (**A**). All infected cells and all infected cells with fragmented GA and cytosolic CPAF were counted, and the relative number of infected cells with cytosolic CPAF as the share of total cells with fragmented GA was calculated (number of infected cells counted: 434, 318). Shown are means of 4 independent experiments ± SEM. (**D**) Quantification of immunogold-labelled CPAF as shown in (**B**). Regions of inclusions or cytoplasm were outlined using ITEM software and the number of gold particles was counted within these regions. Uninfected but stained cells were used as negative control.

Immunofluorescence experiments identified cytosolic CPAF most often at one pole of the inclusion during acute infection, a staining pattern commonly reported for CPAF (Fig. 5A). In persistent infection, the number of cells with cytosolic CPAF-staining was reduced but CPAF was still found around the inclusion in about 10–15% of cells (Fig. 5A and data not shown). A similar experiment was performed with YFP-Golgi-HeLas to quantify the cytosolic presence of CPAF with respect to fragmentation of the GA. The results show that in acute infection 62% of the infected cells with fragmented GA displayed cytosolic CPAF; with PenG treatment this rate dropped to 14.4% (Fig. 5C).

Immune-EM confirmed these findings (Fig. 5B). CPAF was found freely in the cytosol in acute infection; in persistent infection the portion of cells where CPAF was detected outside the inclusion was strongly reduced but some cells were still identified where CPAF was cytosolic (Fig. 5B). Quantification of this labelled CPAF shows that there are significantly more immune-gold particles in the cytosol of acutely infected cells in comparison to PenG-treated infections (Fig. 5D).

CPAF therefore appears to be made at lower levels also during PenG-treatment of *C. trachomatis*-infected cells but secretion into the cytosol is strongly reduced, as already shown for other situations of chlamydial persistence. Since there is thus no detectable CPAF in the cytosol of most persistently infected cells but Golgi-fragmentation and sphingomyelin-transport into the inclusion are not reduced, it seems clear that the cytosolic CPAF is not required for these events.

## Discussion

Many of the known cell biological effects of chlamydial infection are not understood down to the molecular level. Genetic modification of *Chlamydia* is, although now in principle possible, still not readily available. *C. trachomatis* mutants have been made especially by random mutagenesis [30] and sometimes to some degree characterized, as was done for a CPAF-deficient mutant recently [14]. When we embarked on this study we therefore used a system of *in vitro* persistence to test the relationship between CPAF-secretion into the cytosol, Golgi-fragmentation and sphingomyelin-transport into the inclusion; a CPAF-deficient mutant was eventually used to confirm some aspects. The results show a co-occurrence of Golgi-fragmentation and sphingomyelin-transport also in persistent infection, compatible with the view that the two events are causally linked. At least at the level of microscopical examination, these two events however appeared independent of cytosolic CPAF. The CPAF-deficient strain induced normal Golgi-fragmentation, confirming recent results [14].

Based on our previous results that ectopic CPAF-expression can cause Golgi-fragmentation [15] we had expected that the presence of cytosolic CPAF correlates with Golgi-fragmentation. This was however clearly not the case, at least not in as far as the detection by immunostaining permits. CPAF thus can induce Golgi-fragmentation but *Chlamydia* can induce Golgi-fragmentation without CPAF, suggesting two independent mechanisms. It is difficult to quantify Golgi-fragmentation, and it is possible that the fragmentation is stronger or even different in some way in the presence of CPAF. Morphological changes to the GA that appear as ‘fragmentation’ are found in many physiological, pathophysiological and experimental circumstances: mitosis [31] (linked to MAP kinase activity [32]), cellular stress such as heat shock [33] or treatment with a nitric oxide scavenger [34] are examples; Golgi-fragmentation is associated with neurodegenerative diseases such as Parkinson’s or amyotrophic lateral sclerosis [35], among others. Golgi-fragmentation occurs upon experimental reduction of GA matrix proteins [36] or overexpression of other proteins (BOK is an example [37]); it has been described in viral infection [38], in infection with *Legionella* [39] and with *Shigella* [40] as well as with *Toxoplasma* [41] (these are only examples). Indeed, we have endeavoured to look at Golgi-fragmentation in *Chlamydia*-infected MEFs but found that the GA had a fragmented appearance even during normal culture in the absence of infection (data not shown).

This variety of stimuli may cause Golgi-fragmentation by one common mechanism triggered by all of them, or by a variety of molecular pathways; presumably, most instances of interference with exocytic vesicular transport can affect GA-integrity. Either way, since many triggers can end up with Golgi-fragmentation it is easily possible that *Chlamydia* generates two separate triggers, one of which may be CPAF.

Sphingomyelin/ceramide-acquisition through interception of post-GA-transport vesicles by the chlamydial inclusion has been described many years ago. Although the evidence is suggestive that Golgi-fragmentation is causally linked to sphingomyelin-uptake and to chlamydial growth it has to be acknowledged that there is no direct proof. The peptide inhibitors WEHD-fmk (developed as a caspase-1-inhibitor) as well as the calpain inhibitor III inhibited both Golgi-fragmentation and chlamydial growth [12]. This may mean that the inhibitors block an essential protease – and WEHD-fmk blocks the effects of CPAF-expression [15] – but it may also be an indirect effect. It is possible that the inhibitors block any activity of *Chlamydia* and thereby block its growth (independently of CPAF or other proteases), and *Chlamydia* may therefore simply not reach the stage where it fragments the GA. The inhibitors may also interfere with cellular vesicular transport and thereby inhibit *Chlamydia*. Knock-down of golgin-84 partly reversed the effect of WEHD-fmk; this may mean that the cleavage of golgin-84 [12] (later disputed to occur in intact cells [13]) is a driving mechanism. However, since golgi-84-KD strongly increases chlamydial growth on its own, it is difficult to separate the effects.

It has to be noted that there are a few remarkable coincidences. CPAF causes Golgi-fragmentation and Golgi-fragmentation occurs during chlamydial infection; however, Golgi-fragmentation during infection appears not to require CPAF. WEHD-fmk blocks CPAF [15] and blocks chlamydial sphingomyelin acquisition and growth [12]; however, at least sphingomyelin acquisition seems to proceed normally without cytosolic CPAF. This may seem extraordinary but nevertheless it is the conclusion supported by the sum of the available evidence.

We believe that our data, by looking at a model of persistent infection, add to the understanding of cell biological events that occur during chlamydial infection. However, substantially more work is likely necessary to understand the function of CPAF, as well as Golgi-fragmentation and sphingomyelin-transport, whether linked to CPAF or not.

## Supporting Information

Table S1 **Contains all raw data of the numerical data shown in the figures.**
(XLSX)
